# Functional Analysis of Alleged NOGGIN Mutation G92E Disproves Its Pathogenic Relevance

**DOI:** 10.1371/journal.pone.0035062

**Published:** 2012-04-18

**Authors:** Julia Zimmer, Sandra C. Doelken, Denise Horn, Jay C. Groppe, Eileen M. Shore, Frederick S. Kaplan, Petra Seemann

**Affiliations:** 1 Berlin Brandenburg Center for Regenerative Therapies, Charité–Universitätsmedizin Berlin, Berlin, Germany; 2 Berlin Brandenburg School for Regenerative Therapies, Charité–Universitätsmedizin Berlin, Berlin, Germany; 3 Institut für Medizinische Genetik und Humangenetik, Charité–Universitätsmedizin Berlin, Berlin, Germany; 4 Department of Biomedical Sciences, Baylor College of Dentistry, Texas A & M Health Science Center, Dallas, Texas, United States of America; 5 Department of Orthopaedic Surgery, Perelman School of Medicine, University of Pennsylvania, Philadelphia, Pennsylvania, United States of America; 6 Center for Research in FOP and Related Disorders, Perelman School of Medicine, University of Pennsylvania, Philadelphia, Pennsylvania, United States of America; Innsbruck Medical University, Austria

## Abstract

We identified an amino acid change (p.G92E) in the Bone Morphogenetic Protein antagonist NOGGIN in a 22-month-old boy who presented with a unilateral brachydactyly type B phenotype. Brachydactyly type B is a skeletal malformation that has been associated with increased Bone Morphogenetic Protein pathway activation in other patients. Previously, the amino acid change p.G92E in NOGGIN was described as causing fibrodysplasia ossificans progressiva, a rare genetic disorder characterized by limb malformations and progressive heterotopic bone formation in soft tissues that, like Brachydactyly type B, is caused by increased activation of Bone Morphogenetic Protein signaling. To determine whether G92E-NOGGIN shows impaired antagonism that could lead to increased Bone Morphogenetic Protein signaling, we performed functional assays to evaluate inhibition of BMP signaling. Interestingly, wt-NOGGIN shows different inhibition efficacies towards various Bone Morphogenetic Proteins that are known to be essential in limb development. However, comparing the biological activity of G92E-NOGGIN with wt-NOGGIN, we observed that G92E-NOGGIN inhibits activation of bone morphogenetic protein signaling with equal efficiency as wt-NOGGIN, supporting that G92E-NOGGIN does not cause pathological effects. Genetic testing of the child's parents revealed the same amino acid change in the healthy father, further supporting that p.G92E is a neutral amino acid substitution in NOGGIN. We conclude that p.G92E represents a rare polymorphism of the *NOGGIN* gene - causing neither brachydactyly nor fibrodysplasia ossificans progressiva. This study highlights that a given genetic variation should not be considered pathogenic unless supported by functional analyses.

## Introduction

NOGGIN (NOG) is a secreted homodimeric protein. The name originates from the observation that high doses of NOG injected into *Xenopus laevis* embryos caused excessive head development [1]. Later it was shown that NOG specifically inhibits activity of Bone Morphogenetic Proteins (BMPs) and Growth and Differentiation Factors (GDFs) with different efficacies [2]–[5]. BMPs were initially identified as potent bone inducers by Marshall Urist [6]. Today it is known that BMP function is not restricted to skeletal development and regeneration, but fulfill essential functions in several non-skeletal organs including brain, heart, liver, lung, kidney and skin [7]. BMPs belong to the TGFβ superfamily and bind extracellularly to a heterotetrameric complex of type I and type II receptors. The signal is transmitted into the nucleus via phosphorylation of signaling molecules like SMADs, where gene transcription is activated. A main mechanism controlling the signaling cascade both spatially and temporarily are extracellular antagonists like NOG. Analysis of the crystal structure of the BMP7/NOG complex indicated that NOG inhibits signal transmission by occluding the receptor binding site [8].

Imbalance between agonists, antagonists and receptors can result in BMP linked disorders. As NOG is especially important in bone development and function, NOG mutations are linked to several skeletal diseases that are characterized either by joint fusions and/or malformations of the phalanges [9]. Specifically, NOG mutations are described to cause proximal symphalangism (SYM1; OMIM #185800), multiple synostosis syndrome (SYNS, OMIM: #186500), tarsal-carpal coalition syndrome (TCC; OMIM #186570), stapes ankylosis with broad thumbs and toes (OMIM #184460), and brachydactyly type B2 (BDB2; OMIM #611377) as recently reviewed by Potti et al. [10]. All of these phenotypes are the result of a misregulated BMP signaling pathway during human skeletal development. BMP signaling is also altered in fibrodysplasia ossificans progressiva (FOP; OMIM #135100), a rare and disabling autosomal dominant disorder characterized by limb malformations and progressive heterotopic bone formation that leads to complete ankylosis of nearly all joints of the axial and appendicular skeleton [11]–[14]. In 2006, Shore and colleagues linked FOP to chromosome 2q23-q24 and identified the underlying genetic cause of FOP: a heterozygous point mutation in the activin A type I receptor gene (*ACVR1*), a BMP type I receptor, in all classically affected individuals worldwide [15]. It was later shown that the identified missense mutation in *ACVR1* at position c.G617A leading to the amino acid change p.R206H is an activating mutation [16].

Previous to the identification of *ACVR1* mutations in FOP, defects in the BMP signaling pathway had been hypothesized to be responsible for FOP as BMPs regulate multiple steps in development and can induce heterotopic osteogenesis [17], [18]. Initially, BMP4 was considered a primary candidate as a disease-causing gene as it is over-expressed in lesions of FOP patients, in lymphoblastoid cells and in highly vascular pre-osseous fibroproliferative cells [19]–[21]. However, linkage analysis excluded chromosome 14, the location of *BMP4*, and no mutations in BMP4 could be found in FOP patients [15], [22], [23]. *NOG*, a potent extracellular BMP antagonist, was also considered a candidate gene for FOP [24], [25] since BMP4 is antagonized by NOG and also up-regulates *NOG* expression in a negative feedback loop [2], [26].

*De novo* mutations in the *NOG* gene in FOP patients were reported, including the same guanine to adenine substitution at nucleotide 275 leading to the amino acid change p.G92E that we identified in our patient [27], [28]. In response to such reports of the involvement of *NOG* mutations in FOP [29]–[31], several studies providing evidence that FOP is not linked to *NOG* mutations have also been reported [32]–[37], establishing an unresolved issue regarding whether NOG mutations can cause FOP.

NOG activity assays have been successfully established to investigate the functional activity of NOG mutations in BDB2 patients [38] and are used in this study to evaluate the p.G92E substitution. Here we provide evidence based on clinical as well as experimental data that the amino acid change G92E in NOG does not impair NOG function but represents a polymorphism of *NOG*.

## Results

### Patient

**Figure 1:**
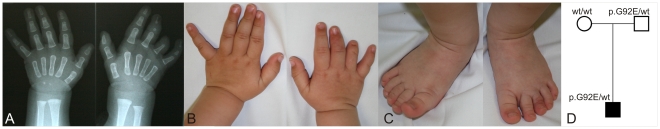
Patient phenotype. X-rays of the patients hands (A) were taken before the operation, at the age of 4 months, showing the unilateral atypical BDB-like phenotype. Photographs of hands (B) and feet (C) were taken after the surgical correction, at the age of 22 months. Note that the middle phalanx of the 3rd left toe was removed and implanted as the middle phalanx of the 3rd finger of the right hand in order to stabilize the digit and improve functionality. The pedigree (D) shows the patient carrying the heterozygous change p.G92E in NOG which he inherited from the healthy father.

The patient of European descent was referred to the department of clinical genetics at the age of 22 months. He presented with unilateral atypical brachydactyly type B-like (BDB-like) of the right hand and a negative family history (**Fig. 1**). Clinically, his right hand showed shortening of the 3^rd^ to 5^th^ digits with a rudimentary finger nail of 4^th^ digit. Missing middle and distal phalanges and a hypoplastic proximal phalanx of the 4^th^ finger as well as hypoplastic middle and distal phalanges of digits III and V were demonstrated by a hand radiograph (**Fig. 1A**). Both clinically and radiologically, the left hand did not show any abnormality. His toes were normal and he had no heterotopic ossification or any other skeletal features of FOP such as fused and malformed cervical vertebrae, osteochondromas of the proximal medial tibias, or short, broad femoral necks [39].

The patient was tested for mutations in two candidate genes for BDB, *ROR2* and *NOG*. Sequencing revealed no mutation in the *ROR2* gene, but a heterozygous substitution c.G275A, p.G92E in the *NOG* gene. Analysis of the healthy parents showed that the unaffected father also carried this substitution. Patient follow-up at the age of 4 years revealed that apart from the unilateral brachydactyly the boy was healthy and did not subsequently show any signs of FOP.

### Structural analysis of p.G92E

**Figure 2:**
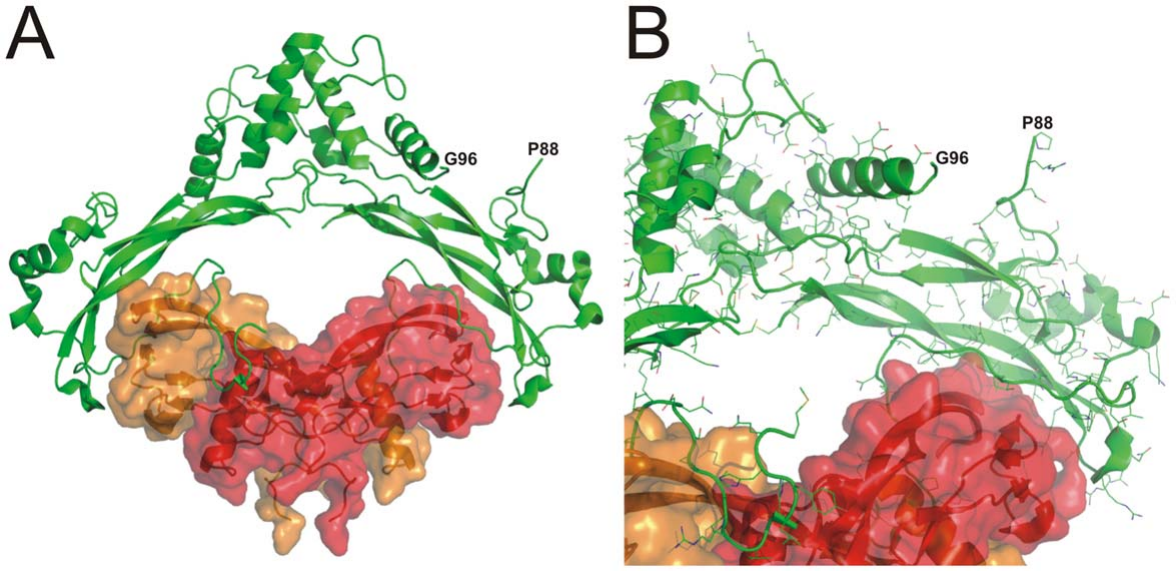
Three-dimensional model of the NOG-BMP7 complex highlighting the unstructured polyglycine loop that harbors the substitution in p.G92E. NOG-BMP7 complex (PDB: 1M4U) is depicted as a cartoon structure, with monomers of the NOG homodimer in dark in light green and monomers of the BMP7 homodimer in red and orange with surfaces depicted (A). Labeled residues flank the polyglycine loop, which is unresolved due to an apparent high flexibility associated with the largely unrestricted chain of residues. The NOG monomers on the left are tilted slightly into, and the BMP monomers slightly out of, the image plane. The complex in the zoomed view (B) is tilted further in the same direction, as well as slightly counter-clockwise about the perpendicular axis.

The single residue substitution in p.G92E is located within an apparently highly flexible segment of the human NOG protein called the polyglycine loop that comprises amino acid residues 89–95 [8]. Due to the apparent lack of uniform structure and thus lack of electron density in the computed maps to guide the process, the polyglycine loop could not be modeled and incorporated into the crystal structure (1M4U) deposited in the PDB (**Fig. 2**). NOG is a dimeric extra-cellular protein that binds to and sequesters the dimeric signal ligands, blocking recruitment of cell surface receptors into a heteromeric signaling complex and transduction of the signal across the membrane. Because the polyglycine loop projects out into the solvent away from the ligand-binding interface located on the opposite surface, direct interactions between the substituted loop and BMP signal ligand are precluded.

### In vitro analysis of G92E-NOG

**Figure 3:**
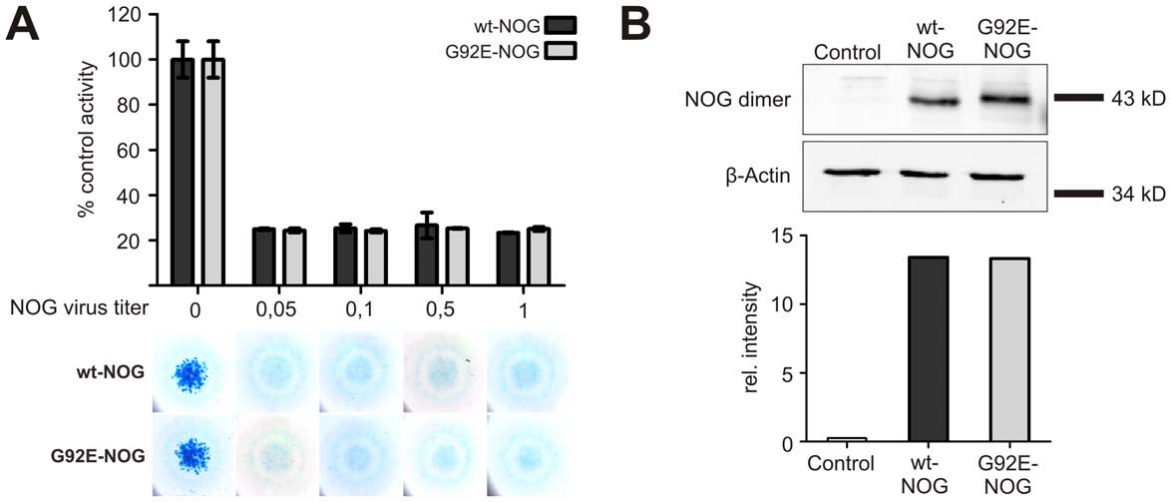
Biological activity of wt-NOG and G92E-NOG and protein production in chicken micromass cells are comparable. Chicken micromass cells were infected with either wt-NOG or G92E-NOG from 1*10e07 viral particles/ml and decreasing to 0.05*10e07 viral particles/ml (A). At day 5, cells were stained with Alcian blue, and dye concentration quantified spectrophotometrically at 595 nm. Non-infected controls were normalized as 100% activity. Data shown are taken from a representative experiment performed with 3 replicates each. Error bars indicate standard deviation. Pellets from cells infected with 1*10e07 viral particles/ml were collected at day 3 from the same chicken micromass experiment to perform Western Blot analysis (B). Uninfected cells were used as a control. After SDS-PAGE under non-reducing conditions and subsequent Western Blot, NOG and β-Actin were detected with specific antibodies. For quantification, NOG was normalized to β-Actin. Wt-NOG and G92E-NOG are expressed in equal amounts in the micromass cultures.

We investigated the G92E-NOG amino acid change in the *in vitro* chicken micromass system to test whether this amino acid change might alter NOG function and potentially cause brachydactyly or FOP. Using a previously established assay for BMP-induced chondrogenesis in micromass cultures [5], we compared the ability of wt-NOG and G92E-NOG to block the natural chondrogenesis of the cultures (**Fig. 3A**). Compared to the uninfected control, both wt-NOG and G92E-NOG inhibited chondrogenesis even when using very low virus titers. To assure that the comparable activity of wt-NOG and G92E-NOG was not due to differences in protein expression levels in the micromass cells, we confirmed via western blot that the amount of wt-NOG and G92E-NOG protein was similar in the cells. The uninfected control contained no detectable NOG protein (**Fig. 3B**).

**Figure 4:**
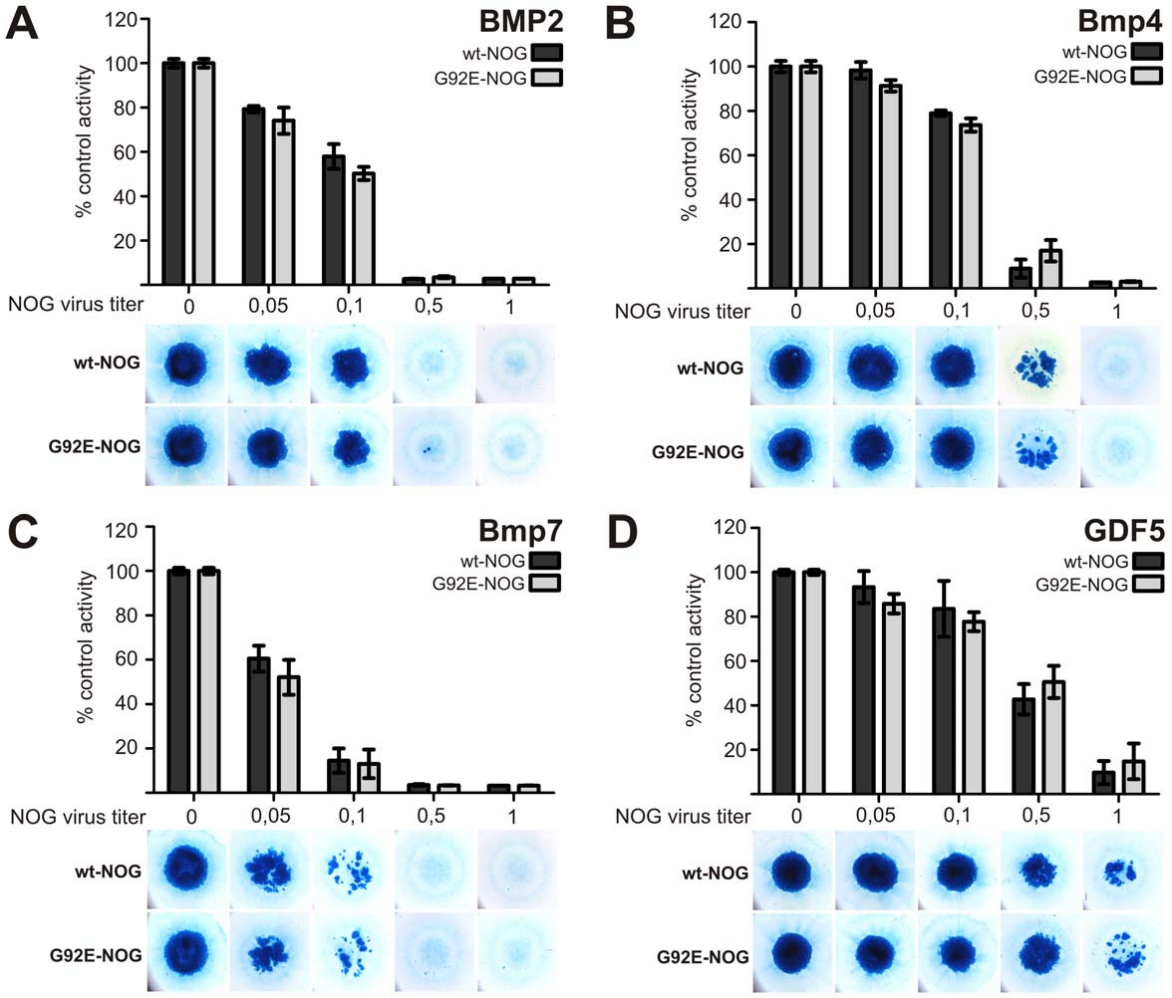
wt-NOG and G92E-NOG show comparable ability to block BMP targets in the chicken Micromass system. Chicken micromass cells were infected with 1*10e07 viral particles/ml containing the gene for BMP2 (A), Bmp4 (B), Bmp7 (C) or GDF5 (D). Co-infection was performed with increasing virus titers of either wt-NOG or G92E-NOG as indicated. At day 5, cells were stained with Alcian blue, and dye concentration quantified spectrophotometrically at 595 nm. Controls infected exclusively with BMPs were normalized as 100% activity. Data shown are taken from a representative experiment performed with 3 replicates each. Error bars indicate standard deviation.

We further investigated the ability of wt-NOG and G92E-NOG to inhibit the activity of a set of BMPs which are co-expressed during limb development [40]. G92E-NOG was able to block all tested BMPs in a comparable, dose dependent manner to wt-NOG (**Fig. 4**). Comparison of the levels of inhibition of the BMPs by NOG showed that Bmp7 had the highest sensitivity. With a NOG titer of only 1/10 of the BMP titer, Bmp7-induced chondrogenesis is nearly completely blocked whereas all other BMPs remain able to induce chondrogenesis at this level of competition. Both BMP2 and Bmp4 were antagonized efficiently by both, wt-NOG and G92E-NOG, though less potently than Bmp7. Of this tested set of BMPs, GDF5 was blocked least efficiently by NOG but was still able to induce Alcian blue positive nodules when NOG and GDF5 titers were equal.

## Discussion

The clinical relevance of *NOG* missense mutations in FOP, a rare but fatal genetic disorder, has been a matter of intense discussion for nearly a decade [32]–[37]. Here we provide evidence based on functional data that the G92E substitution in the NOG protein does not cause FOP but rather represents a rare neutral genetic polymorphism.

We identified G92E-NOG in a 22-month-old patient with an atypical unilateral brachydactyly phenotype and in his healthy father. Brachydactylies represent a group of skeletal disorders characterized in general by shortened digits in hand and feet due to abnormalities in the developmental process of phalanges or metacarpals/metatarsals [41]. Brachydactyly type B1 (BDB1; OMIN #113000) is caused by truncating mutations in the *ROR2*, whereas BDB2 is the result of mutations in *NOG*. As the patient described here presented with unilateral atypical BDB-like phenotype, sequencing of *ROR2* and *NOG* was performed, revealing the nucleotide substitution c.G275A in the *NOG* gene resulting in the amino acid change p.G92E. A search through the Human Gene Mutation Database indicated that G92E-NOG is currently not associated with any type of brachydactyly but with FOP.

In 2000, Lucotte and colleagues reported a genetic linkage between *NOG* and FOP, whereas Xu et al. excluded such a linkage between *NOG* and FOP and could not identify mutations of the *NOG* gene in a large cohort of FOP patients [35], [42]. In addition, three novel mutations in *NOG* published by Semonin et al. were subsequently challenged to be technical PCR errors due to the use of a nested PCR approach [28], [36]. Consequently, the necessity to present photographs and radiographs of the studied FOP patients has been emphasized to assure the correct clinical diagnosis and that the same phenotypes are compared [34]. Upon identification of heterozygous missense activating mutations in *ACRV1* as the genetic cause of FOP in 2006, additional questions regarding the validity of NOG mutations in FOP were raised [15], [27], [30], [33], [37].

The nucleotide substitution c.G275A in the *NOG* gene leading to p.G92E was described for two patients with FOP of Spanish origin. Of note, one of the patients was also positive for the p.R206H mutation in *ACVR1*, raising the question of the likelihood that two “pathogenic” mutations causing a genetic disorder as rare as FOP would be found in a single patient [27], [28].

Our patient with unilateral atypical BDB-like phenotype and his healthy farther were positive for the amino acid change c.G275A, p.G92E in the *NOG* gene previously described to be a cause of FOP [27], [28]. Neither of these two individuals, the now 4-year-old boy or his 42-year-old father, showed any clinical features of FOP whatsoever.

From a structural standpoint, the substitution in p.G92E is situated in the most flexible portion of the NOG protein, a segment not interpretable from the electron density maps of the 3D-structure determination [8] (**Fig. 2**). Unlike much of the polypeptide of NOG proteins that is required for folding, stability and complex formation, the polyglycine segment is not conserved among different species. For example, in dogs the loop is expanded yet in non-mammals is completely absent [43]. Moreover, the residue at position 92 is distal to and oriented away from the ligand-binding interface, consistent with the neutral effect of the substitution on function as an antagonist.

Further, using experimental assays for BMP signaling, we did not identify differences between wild-type and mutant NOG protein to inhibit control chicken micromass cells (**Fig. 3**). This observation is in contrast to disease causing NOG mutations like P35R or R167G, where chicken micromass controls are shown to be less efficiently blocked compared to the wt-NOG [38]. However, in the same publication of Lehmann et al. some BDB causing NOG mutations did also not show a loss of inhibitory activity on chicken micromass control cells, like A36P and P187S. Hence we expanded our approach to compare the ability of G92E-NOG and wt-NOG to block chondrogenesis in chicken micromass cells expressing BMPs/GDFs known to be essential in limb development. We showed that BMP2, Bmp4, Bmp7 and GDF5 are blocked by wt-NOG and G92E-NOG comparably in a dose dependent manner (**Fig. 4**). As the tested BMPs are all known to be crucial players during limb patterning, changes in NOG affinity for these BMPs could have provided an explanation for the hand phenotype of the patient [5], [40]. It should be noted, that in contrast to previous data, Bmp7 was antagonized very efficiently by NOG when compared to BMP2, Bmp4 and GDF5 [2]. Furthermore, NOG had been hypothesized to cause FOP through decreased antagonism of BMP4 since this BMP was found to be over-expressed in lesions of FOP patients [20]. However, if a mutation in NOG resulted in FOP due to decreased BMP4 antagonism, this would have been readily apparent in the chicken micromass system (**Fig. 4**). As there is no difference in Bmp4 antagonism comparing wt-NOG and G92E-NOG, we can exclude this path of pathogenicity. The general applicability of chicken micromass cultures for the evaluation of FOP causing mutations has been demonstrated as mutations in ACVR1 do result in changes in BMP signaling which can be monitored in this system [16].

The absence of differences between G92E-NOG and wt-NOG in our functional tests supports that the observed unilateral atypical BDB-like phenotype in our patient is not due to the polymorphism in *NOG*. His malformation is more likely caused by an isolated local disruption of embryonic vessels during early development. Polygenic or multifactorial developmental disturbances rather than single gene germline mutations are thought to be the cause of many unilateral limb defects, especially with respect to hereditary monogenic types of brachydactylies as these generally manifest bilaterally [44]. Furthermore, empirical data support our interpretation. In the exome sequence variant database of the Seattle University (http://evs.gs.washington.edu/EVS) 25 of 10129 alleles (23 of European American origin; 2 of African American origin) are listed containing the G92E exchange [45]. This implies a population allele frequency of 1/400. As both FOP and BDB are rare genetic disorders, it is very unlikely that G92E-NOG causes either of these diseases. We conclude that the G92E change in NOG is a polymorphism neither causing BDB nor FOP.

Our analysis raises important issues with respect to genetic counseling. Since the G92E amino acid change in NOG is reported to be pathogenic for FOP in many databases, such as the Human Gene Mutation Database, parents could be wrongly informed that their child has a FOP causing mutation when, in fact, a neutral polymorphism has been identified. In our opinion it is imperative that this fact becomes common knowledge and noted in the relevant database, and further that other *NOG* mutations described to cause FOP are evaluated through experimental tests. The necessity of such functional assays for the evaluation of putatively disease causing mutations was recently underlined by several studies. For example, genome information of Dr. James Watson by next-generation sequencing technology identified 20 mutations associated with increased disease risks without becoming manifest in the apparently healthy carrier [46], [47]. This notion was also supported in a larger scale by MacArthur et al. who showed that healthy humans carry a high number of putative complete loss-of-function mutations in protein-coding genes without phenotypic consequences, suggesting an unexpected high degree of redundancy in the human genome [48]. It is predicted that for the majority of human genes a single functional allele is sufficient to exert the normal function [49]. Considering these results, functional assays are indispensable to analyze the potential pathogenicity of a mutation.

## Materials and Methods

### Ethics statement

Clinical investigations have been conducted according to the principles expressed in the Declaration of Helsinki. Ethical approval of the study was given by the ethical board Charité. Written informed consent for genetic testing was received from all analyzed individuals or by parents on behalf of their child.

### Molecular Analysis of patient and parents

Genomic DNA was extracted from peripheral blood samples by standard methods. The coding regions of tyrosine kinase-like orphan receptor 2 gene (*ROR2*) and *NOG* as well as the flanking intronic sequences were amplified by standard PCR protocols. The primer sequences and PCR conditions for the molecular testing were previously published (for *NOG*: [38]; for *ROR2*: [50]). PCR products were analyzed on 2% agarose gels. Sequencing was done using the ABI Prism BigDye Terminator Sequencing Kit (Applied Biosystems, Foster City, CA, USA) with PCR primers used as sequencing primers. Products were evaluated on an automated capillary sequencer (Applied Biosystems 3730, Foster City, CA, USA). Identified sequence changes were evaluated using dbSNP135 and the Human Gene Mutation Database (HGMD) (https://portal.biobase-international.com/hgmd) as a reference.

### NOG-BMP7 complex

The image of the three-dimensional structure of NOG-BMP7 complex was produced from the PDB-file 1M4U [8] rendered by PyMOL Molecular Graphics System, Version 1.2r3pre, Schrödinger, LLC.

### Virus preparation

Mouse *Bmp4* in RCAS-A was provided by Pip Francis-West [51], human *GDF5* in RCAS-A was previously described [52]. Coding sequences of human *BMP2*, mouse *Bmp7* and human *NOG* were amplified by PCR and cloned into the shuttle vector pSLAX-13. *In vitro* mutagenesis for human *NOG* was performed with the QuickChange Site-Directed Mutagenesis Kit (Agilent Biotechnologies, Santa Clara, CA, USA) BMP2, GDF5 and Bmp7 were cloned into RCAS(BP)A. Wild-type (wt)-*NOG* and G92E-*NOG* were cloned into RCAS(BP)B to allow co-expression of NOG with different BMPs. Cloning into retroviral vectors, production of viral supernatant in DF1 cells and concentration of viral particles was performed as described previously [53]. In short, DF1 cells were transfected with RCAS retroviral vectors and supernatant harvested at 3 consecutive days. Viral particles were concentrated via ultra-centrifugation, followed by determination of viral titer through infection of DF-1 cells (ATCC: UMNSAH/DF-1 #CRL-12203) and counting of cells positive for an RCAS specific antibody.

### Chicken Micromass Culture System

Chicken micromass cultures were primary isolated from limb buds of day 4.5 chicken embryos and performed as previously described [38]. Cells were plated at a density of 2×10e05 cells/14 µl-drop. Single and co-infections were performed with concentrated viral supernatants adjusted to 1×10e07 infectious units/ml. To evaluate chondrogenesis, micromass cultures were fixed and stained with 0.05% Alcian blue. Alcian blue staining was quantified after extraction with 6 M guanidine-HCl and spectrophotometically measured at 595 nm.

### Western Blotting

Western Blot analysis was performed as previously described [38] with minor changes: Micromass cells were harvested at day 3 and lysed in lysis buffer (50 mM HEPES, 50 mM NaCl, 10 mM EDTA, 10% glycerol, 1% Triton, 100 mM PMSF). Immunodetection was performed using an anti-NOG antibody (sc-25656, Santa Cruz Biotechnology, Santa Cruz, CA, USA), and an anti-Actin antibody (A5441, Sigma-Aldrich, St.Louis, MO, USA) as primary antibodies. Signals were detected via IRDye labeled secondary antibodies (IRDye goat anti rabbit 800; IRDye goat anti mouse 680, LICOR, Lincoln, NE, USA) and quantified using the Odyssey infrared imaging system (LICOR).
